# Prevalence of HIV and HCV among injecting drug users in three selected WHO-EMRO countries: a meta-analysis

**DOI:** 10.1186/s12954-021-00505-4

**Published:** 2021-05-27

**Authors:** Shah Jahan Shayan, Rajab Nazari, Frank Kiwanuka

**Affiliations:** 1 Department of Fundamental of Nursing, School of Nursing, Kabul University of Medical Sciences, Jamal Mina, 3rd District, Kabul, Afghanistan; 2 Department of Nursing Sciences, University of Eastern Finland, Kuopio, Finland

**Keywords:** HIV, Hepatitis C, Injecting drug users, Iran, Afghanistan, Pakistan

## Abstract

**Background:**

HIV and Hepatitis C Virus (HCV) infections are responsible for a significant burden of mortality and morbidity, particularly in developing countries. This study sought to determine the prevalence of HIV and Hepatitis C among injecting drug users in Afghanistan, Iran, and Pakistan.

**Methods:**

This review conforms to the Preferred Reporting Guidelines for Systematic Reviews and Meta-Analysis (PRISMA) statement. Databases including PubMed, Scopus, Web of Science/Knowledge, SID.ir, and MAGIRAN were searched. Studies that were published from 2003 up to 2018 were considered for analysis. Studies were screened for inclusion in duplicate, and also, that data were narratively synthesized.

**Results:**

We report on data from 79 articles. The total number of participants in studies that assessed the prevalence of HIV among injecting drug users included 68,926 participants, while those from studies that assessed HCV prevalence were 23,016 participants. Overall HIV and HCV prevalence among injecting drug users in the three selected countries were 9.1% (95% CI 6.9–12.0%) and 48.3% (95% CI 43.9–52.7%), respectively. Iran had the highest HIV prevalence of 11.0% among injectable drug users (95% CI 8.4–14.2%), while Afghanistan had the lowest HIV prevalence of 3.1% (95% CI 1.5–6.3%) among three selected countries. In Pakistan, the prevalence of HIV was 8.6% (95% CI 4.8–15.0%). Regarding HCV prevalence, Pakistan had the highest while Afghanistan had the lowest, 54.4% (95% CI 33.5–73.9%) and 37.3% (95% CI 35.2–39.4%), respectively. HCV prevalence in Iran was 47.7% (95% CI 43.4–52.0%).

**Conclusion:**

Injecting drug users form a special cohort of persons at risk of HIV and Hepatitis C infections. The prevalence of HIV and Hepatitis noted from our findings is significantly high. Awareness of the grave risk of spreading HIV and Hepatitis C associated with sharing needles is recommended among this sub-group of drug users.

## Background

HIV and Hepatitis C Virus (HCV) infections are among the most significant public health challenges globally. They bear a significant burden of mortality and morbidity, particularly in developing countries. In 2017, WHO reported that 36.9 million people were living with HIV globally with record of 940,000 deaths [1]. HIV suppresses the immune system, in so doing, it makes the infected person susceptible to infectious diseases that may lead to death [2]. HIV can also invade the central nervous system that leads to severe neurological problems [3]. With regard to HCV, there are 177.5 million infected adults globally and up to 0.5 million deaths every year. Hepatitis due to HCV has the potential to become chronic, consequently leading to cirrhosis of the liver which may cause liver cancer and death [4]. Both HIV and HCV can be spread through body fluids. Certain behaviors like sharing injection equipment can lead to transmission of such pathogens[3, 5].

Estimates have indicated that, in Afghanistan, 5900 people living with HIV, in Iran and Pakistan 60,000 and 150,000 people living with HIV, respectively [1]. The prevalence of HCV in Afghanistan according to a systematic review in 2015 was estimated to be 0.7% among the general population [6]. In Pakistan and Iran, the prevalence of HCV among the general population was 4.8% and 0.3%, respectively [7, 8]. HCV and HIV are heavily associated with injecting drug users (IDUs) are highly susceptible to transmission of HIV and HCV through sharing sharp materials such as infected needles/syringes[9].

Globally, there are approximately 13 million IDUs; of these, estimates have shown that 1.7 million are infected with HIV[1, 10]. In addition, approximately 10% of HIV infections are transmitted through sharing materials during injectable drug usage. Concerning HCV, the prevalence of HCV among injecting drug users is estimated at 67% globally. Co-infection of HCV and HIV accounts for about 2.2 million people, with more than half of these being among IDUs [1].

Furthermore, Aceijas and colleagues (2007) revealed that about 50% of IDUs were HCV positive in 49 countries. Prevalence of HCV was reported with significant variance from 2% up to 100% [11]. There was no study assessing the extent of HIV and HCV among IDUs in the WHO-EMRO region to the best of our knowledge. Needless to say, this region has profound significance concerning drug production and usage. In fact, Afghanistan is the leading producer of opium in the world [12]. This does not only influence drug usage; it has a fundamental role in distribution chains to other countries, especially those in the neighborhood, particularly Pakistan and Iran.

Moreover, decades of political instability in this region have caused millions of people to migrate to Iran and Pakistan. This said trafficking of drugs goes hand in hand with the movement of people between borders. This can increase the risk of transmission of disease between countries specifically among IDUs relative to other regions of the world.

Prevention efforts among IDUs which focus only on individual behavior modification are likely to result in only a partial decrease in HIV and HCV transmission. To tackle this problem effectively, there is a need for regional interventions at the macro-level. In order to have a collaborative effort toward controlling HIV and HCV among IDUs in an effective manner, baseline data are needed. To serve this goal, we performed a systematic review of studies in these three countries.

### Review Question

What is the prevalence of HIV and HCV among IDUs in Afghanistan, Pakistan, and Iran?

## Methods

*Protocol* This review conforms to the Preferred Reporting Items for Systematic Reviews and Meta-Analysis (PRISMA) statement (Moher, 2009). To ensure that there was no similar work to ours, we did a preliminary scoping search in the International prospective registers of systematic reviews (PROSPERO), Cochrane Library, and Google scholar. The search was done on 1/12/2020.

*Eligibility criteria* Studies that reported the prevalence of HIV and HCV among injecting drug users published in English and Persian languages in peer-review Journals from 2003 up to 2018 in Afghanistan, Pakistan, and Iran were included (see Table 1).

**Table 1:** Inclusion criteria applied to selected articles

Inclusion criteria
The study reported on the prevalence of HIV and/or HCV
The sample was injecting drug users
Samples were tested for antibodies to HIV and HCV by enzyme-linked immunosorbent assay
The study that was published in English or Persian
The study that was conducted in either Afghanistan, Pakistan, or Iran
The study was an empirical study published in a peer-review journal
The study was published from 2003 up to 2018

*Databases* Articles were searched in PubMed, Scopus, Web of Science, Embase, SID.ir, and MAGIRAN.

*Search strategy* Keywords included “Human Immunodeficiency Virus” “Hepatitis C Virus,” “Substance abuse,” “Injecting Drug User,” “Injecting Drug Abuser,” “Intravenous Drug Abuse” “Drug Misuse,” “Drug Abuse,” “Drug Dependence,” “Afghanistan,” “Pakistan,” and “Iran” from 2003 up to 2018 (Table 2). The reference lists of the selected articles were also hand-searched to find additional relevant studies.

**Table 2:** Search terms syntax

Database	Synthix	No
Scopus	( TITLE-ABS-KEY ( human AND immunodeficiency AND virus) AND TITLE-ABS-KEY ( hepatitis AND c) AND TITLE-ABS-KEY ( substance AND abuse) OR TITLE-ABS-KEY ( injecting AND drug AND user) OR TITLE-ABS-KEY ( injecting AND drug AND abuser) OR TITLE-ABS-KEY ( intravenous AND drug AND abuse) OR TITLE-ABS-KEY ( drug AND misuse) OR TITLE-ABS-KEY ( drug AND abuse) OR TITLE-ABS-KEY ( drug AND dependence)) AND DOCTYPE ( ar) AND PUBYEAR > 2002 AND PUBYEAR < 2019 AND ( LIMIT-TO ( AFFILCOUNTRY, "Iran") OR LIMIT-TO ( AFFILCOUNTRY, "Pakistan") OR LIMIT-TO ( AFFILCOUNTRY, "Afghanistan"))	99
Embase	('human immunodeficiency virus infection':ab,ti AND 'hepatitis c virus':ab,ti AND 'substance abuse':ab,ti OR 'injection drug user':ab,ti OR 'injecting drug abuser':ab,ti OR 'intravenous drug abuse':ab,ti OR 'drug misuse':ab,ti OR 'drug abuse':ab,ti OR 'drug dependence':ab,ti) AND afghanistan:ab,ti AND iran: ab,ti AND pakistan: ab,ti AND [2003–2018]/py	309
Web of Sciences	(human immunodeficiency virus) *AND* **TOPIC:**(hepatitis C) *AND* **TOPIC:** (substance abuse) *OR* **TOPIC:** (injecting drug user) *OR* **TOPIC:** (injecting drug abuser) *OR* **TOPIC:** (intravenous drug abuse) *OR* **TOPIC:** (drug misuse) *OR***TOPIC:** (drug dependence) **Refined by:** **COUNTRIES/REGIONS:** ( AFGHANISTAN OR PAKISTAN OR IRAN) **Timespan:** 2003–2018.**Indexes:** SCI-EXPANDED, SSCI, A&HCI, CPCI-S, CPCI-SSH, BKCI-S, BKCI-SSH, ESCI, CCR-EXPANDED, IC	620
PubMed	(("HIV"[Mesh]) AND "Hepatitis C"[Mesh]) AND "Substance Abuse, Intravenous"[Mesh] Filters: From 2003/01/01 to 2018/12/31, Humans, English, Persian	100

*Study selection* We created an endnote (version X.7) Library to store and manage the references. Two reviewers independently search for the articles compared to their articles found and always reached consensus on studies to exclude or include based on the inclusion criteria described above.

*Data collection process* For articles that met the inclusion criteria, information was extracted and recorded in piloted data set in an excel spreadsheet. For included studies, we assessed the study findings' main outcome, including the prevalence of HIV or HCV. Selected articles were kept for future narrative, and excluded articles were also kept in a separate file for future reference was appropriate. The following items were extracted from studies: author, country, year, study design, setting, sample size, and HIV or HCV prevalence.

*Risk of bias in individual studies* The studies were appraised based on the selection criteria (Table 1) and the Joanna Briggs Institute (JBI) critical appraisal tool for systematic reviews checklist for prevalence studies [13]. This tool is a rating list with nine criteria, which can be answered as yes (coded as 1), no (coded as 0), not applicable (coded as NA), or unclear (coded as?); thus, the score for each study ranged from 0 to 9. Depending on its score, we rated each study as low risk [7–9], moderate risk [4–6], or high risk of bias [1–3].

*Data analysis* We used a Random Effects model to estimate the pooled prevalence. The result was displayed in a forest plot and shown high heterogeneity. Our review resulted in 83 peer-review articles from three countries concentrated on HIV and HCV prevalence. Data were reported as the proportion of the infected numbers among total injecting drug users. Prevalence from each article was collected in the form of a table (Table 3, 4, and 5) and then inserted in Comprehensive Meta-Analysis (CMA) version 3. The pooled prevalence of HIV and HCV was calculated with a 95% confidence interval and stratified by country.

**Table 3:** Characteristics of included studies on HIV

Study	Country		Year of Publication		Design	Setting		Sample size		HIV Prevalence		JBI Score
Rahimi-Movaghar et al. [16]	Iran		2009		Cross-sectional	Treatment center and community		899 (F = 38)		10.7%		8
Khajehkazemi et al.[17]	Iran		2013		Cross-sectional	Facilities center		2290		15.2%		7
Javadi et al.[18]	Iran		2013		Cross-sectional	Drop in center		539		1.1%		9
Imani et al.[19]	Iran		2008		Cross-sectional	Rehabilitation center		133		0.8%		7
Zamani et al. [20]	Iran		2006		Cross-sectional	Drop in center and community		207		23.2%		7
Khani et al. [21]	Iran		2003		Cross-sectional	Prison		346		1.2%		7
Mirahmadizadeh et al. [22]	Iran		2009		Cross-sectional	Harm reduction centers		936 (F = 60)		20.5%		9
Davoodian et al. [23]	Iran		2009		Cross-sectional	Prison		249		15.1%		9
Hosseini et al. [24]	Iran		2010		Cross-sectional	Detention center		417		24.4%		7
Malekinejad et al.[25]	Iran		2015		Cross-sectional	Drop in centers and hospital		548		26.6%		9
Nikkhooy el al.[26]	Iran		2012		Cross-sectional	Hospital		205		18.5%		9
kazerouni et al. [27]	Iran		2009		Cross-sectional	Community		360		24.7%		7
Sarveqad et al. [50]	Iran		2005		Cross-sectional	Hospital		53		5.6%		7
Aminzadehv et al. [28]	Iran		2007		Cross-sectional	Hospital		70		30%		7
Moradi et al. [29]	Iran		2012		Cross-sectional	Prison		118		4.2%		6
Khorvash et al. [49]	Iran		2009		Cross-sectional	hospital		92		9.7%		5
Kheirandish et al.[30]	Iran		2010		Cross-sectional	Detention center		459		24.4%		9
Zamani et al. [31]	Iran		2005		Cross-sectional	Treatment center		165		15.2%		8
Ramezani et al.[32]	Iran		2014		Cross-sectional	Clinic		100		19%		6
Sofian et al.[33]	Iran		2012		Cross-sectional	Detention center		153		5.9%		6
Rahbar et al. [51]	Iran		2004		Cross-sectional	Prison and community		101		7%		5
Zamani et al. [52]		Iran	2010	Cross-sectional			Community		118 (F = 3)		0.7%	6
Taghizadeh et al. [53]	Iran		2014		Cross-sectional	Homeless		3044		3.7%		8
Ghasemian et al [54]	Iran		2011		Cross-sectional	Hospital		88 (F = 1)		18.2%		7
Alavi et al. [55]	Iran		2012		Cross-sectional	Prison and Treatment center		109		47.7%		6
Dibaj et al. [56]	Iran		2013		Cross-sectional	Prison		970		6.4%		9
Ilami et al. [57]	Iran		2012		Cross-sectional	Community		158		9.9%		6
Alizadeh et al. [58]	Iran		2005		Cross-sectional	Prison		149		0.6%		9
Mir-Nasseri et al. [59]	Iran		2011		Cross-sectional	Prison and rehabilitation center		458		15.5%		7
Sharif et al. [60]	Iran		2009		Cross-sectional	Hospital		200 (F = 23)		1.5%		7
Khodadadizadeh et al. [61]	Iran		2006		Cross-sectional	Drop in Center		31		9.7%		9
Hashemipour et al. [62]	Iran		2013		Cross-sectional	Community		1599		1.5%		7
Sharifi-Mood et al. [63]	Iran		2006		Cross-sectional	Hospital		31 (F = 1)		25.8%		7
Alipour et al. [36]	Iran		2013		Cross-sectional	Drop-in-center		226		9.4%		6
Alavi et al. [46]	Iran		2007		Cross-sectional	Hospital		154		67.53%		9
Eskandarieh et al. [68]	Iran		2013		Cross-sectional	Rehabilitation center		402		18.8%		6
Honarvar et al. [74]	Iran		2013		Cross-sectional	Counseling center		233		7.7%		7
Alinaghi et al. [80]	Iran		2017		Cross-sectional	Prison		851		8.3%		8
Kuo et al. [34]	Pakistan		2006		Cross-sectional	Harm reduction center		351		0%		8
Emmanuel et al. [35]	Pakistan		2009		Cross-sectional	Community		400		51.3%		9
Achakzai et al.[37]	Pakistan		2007		Cross-sectional	Community		50		24%		3
Bokhari et al. [64]	Pakistan		2007		Cross-sectional	Community		799		11.9%		7
Platt et al. [65]		Pakistan	2008	Cross-sectional			Community		404		2%	7
Khanani et al. [66]	Pakistan		2010		Cross-sectional	Clinic		20		10%		5
Emmanuel et al. [67]	Pakistan		2013		Cross-sectional	Community		46,351		37.8		9
Ilyas Jat et al. [69]	Pakistan		2018		Cross-sectional	Hospital		280 (F = 24)		3.2%		8
Abbasi et al. [70]	Pakistan		2009		Cross-sectional	Hospital		300		0.3%		7
Parviz et al. [81]	Pakistan		2006		Cross-sectional	Community and rehabilitation center		231		0.4%		6
Altaf et al. [82]	Pakistan		2007		Cross-sectional	Harm-reduction program		161		0.6%		8
Akram et al. [83]	Pakistan		2017		Cross-sectional	Community		200		47%		7
Ruisenor Escudero et al. [71]	Afghanistan		2014		Cross-sectional	Community		548		7.1%		7
Nasir et al.[9]	Afghanistan		2010		Cross-sectional	Community		623		1.8%		7
Todd et al.[12]	Afghanistan		2011		Cross-sectional	Community		483		2.1%		7
Todd et al. [38]	Afghanistan		2007		Cross-sectional	Clinic		464		3%		8

**Table 4:** Key characteristics of included studies on HCV

Study	Country		Year of Publication	Design			Setting	Sample size	HCV prevalence		JBI Score
Rahimi-Movaghar et al. [16]	Iran		2009	Cross-sectional			Treatment center and community	899 (F 38)	34.5%		8
Alavi et al. [39]	Iran		2010	Cross-sectional			Hospital	333	30.9%		8
Mir-nasseri et al. [40]	Iran		2011	Cross-sectional			Prisons and rehabilitation centers	518 (F = 54)	69.5%		7
Imani et al.[19]	Iran		2008	Cross-sectional			Rehabilitation center	133	11.3%		7
Khani et al.[21]	Iran		2003	Cross-sectional			Prison	346	47.7%		7
Mir-Nasseri et al. [41]	Iran		2005	Cross-sectional			Prison and drop in center	467	66%		7
Kaffashian et al. (42	Iran		2010	Cross-sectional			Prison	951	42%		8
Ataei et al.[43]	Iran		2010	Cross-sectional			Prison and drop in center	1485	43.4%		7
Nikkhooy et al. [26]	Iran		2012	Cross-sectional			Hospital	154	42.2%		8
Esmaeili et al. [44]	Iran		2012	Cross-sectional			Community and drop in center	895	34.5%		7
Nokhodian et al. [46]	Iran		2012	Cross-sectional			Drop in center	531	47.1%		7
Sarveqad et al. [50]	Iran		2005	Cross-sectional			Hospital	53	67.9%		7
Aminzadehv et al. [28]	Iran		2007	Cross-sectional			Hospital	70	36%		7
Ilami et al.[57]	Iran		2012	Cross-sectional			Community	158	42.4%		6
Khorvash et al. [49]	Iran		2009	Cross-sectional			Hospital	92	57.6%		5
Mirahmadizadeh et al. [22]	Iran		2009	Cross-sectional			Harm reduction center	936 (F = 60)	43.4%		9
Davoodian et al. [23]	Iran		2009	Cross-sectional			Prison	249	64.8%		8
Hosseini et al. [24]	Iran		2010	Cross-sectional			Detention center	417	80%		7
Kheirandish et al.[30]	Iran		2009	Cross-sectional			Rehabilitation center	454	80%		8
Zamani et al. [20]	Iran		2007	Cross-sectional			Drop in center and community	202	52%		6
Ramezani et al. [32]	Iran		2014	Cross-sectional			Clinic	100	56%		6
Sofian et al. [33]	Iran		2012	Cross-sectional			Detention center	153	59.5%		6
Rahbar et al. [51]	Iran		2004	Cross-sectional			Community and Prison	101	59.4		5
Ghasemian et al. [54]	Iran		2011	Cross-sectional			Hospital	88 (F = 1)	37.5%		7
Amiri et al. [72]	Iran		2007	Cross-sectional			Prisoners	81	88.9%		7
Nobari et al. [73]	Iran		2012	Cross-sectional			Community	1747 (F = 14)	34%		7
Alizadeh et al. [58]	Iran		2005	Cross-sectional			Prison	149	31.5%		9
Alavi et al. [75]	Iran		2009	Retrospective study			Document	142 (F = 12)	52.1%		6
Sharif et al. [60]		Iran	2009		Cross-sectional	Hospital		200 (F = 23)		12%	6
Khodadadizadeh et al. [61]	Iran		2006	Cross-sectional			Drop in Center	31	25.8%		8
Sharifi-Mood et al. [63]	Iran		2006	Cross-sectional			Hospital	31 (F = 1)	22.7%		7
Zamani et al. [52]	Iran		2010	Cross-sectional			Community	118 (F = 3)	59.4%		6
Kassaian et al. [76]	Iran		2012	Cross-sectional			Prison	1943 (F = 5)	41.6%		9
Sharhani et al. [84]	Iran		2017	Cross-sectional			Drop-in-center	606	54.8%		7
Rezaie et al. [85]	Iran		2016	Cross-sectional			Drop-in-center	410	42%		7
Honarvar et al. [74]	Iran		2013	Cross-sectional			Counseling center	233	40.3%		7
Eskandarieh et al. [68]	Iran		2013	Cross-sectional			Rehabilitation center	402 (F = 16)	65.9%		6
Alipour et al. [36]	Iran		2013	Cross-sectional			Drop-in-center	226	38.6%		5
Alavian et al. [86]	Iran		2013	Cross-sectional			Treatment center	259 (F = 4)	50%		5
Ataei et al. [87]	Iran		2011	Cross-sectional			Community	136	19.8%		5
Keramat et al. [88]	Iran		2011	Cross-sectional			Counseling center	199	63.3%		8
Mir-Nasseri et al. [89]	Iran		2008	Cross-sectional			Prison and rehabilitation center	518 (F = 54)	59.5%		8
Moradi et al. [90]	Iran		2018	Cross-sectional			Prison	678	42.5%		9
Rehman et al.[47]	Pakistan		2011	Cross-sectional			Community	200	24%		5
Kuo et al.[34]	Pakistan		2006	Cross-sectional			Harm reduction center	351	88%		7
Akhtar et al. [48]	Pakistan		2016	cross-sectional			Community	241	36.1%		5
Achakzai et al. [37]	Pakistan		2007	Cross-sectional			Community	50	60%		3
Butt et al. [77]	Pakistan		2010	Cross-sectional			Prison	76	84.2%		6
Platt et al. [65]	Pakistan		2008	Cross-sectional			Community	404	14.9%		7
Khanani et al. [66]	Pakistan		2010	Cross-sectional			Clinic	20	35%		5
Ilyas Jat et al. [69]	Pakistan		2018	Cross-sectional			Hospital	280 (F = 24)	16.8%		7
Rehan et al. [78]	Pakistan		2009	Cross-sectional			Community	779	89.3%		9
Ali et al. [79]	Pakistan		2011	Cross-sectional			Clinic	42	14.28%		5
Abbasi et al. [70]	Pakistan		2009	Cross-sectional			Hospital	300	44.7%		6
Altaf et al. [86]	Pakistan		2007	Cross-sectional			Harm-reduction program	161	94.3%		7
Waheed et al. [91]	Pakistan		2017	Cross-sectional			Community	100 (F = 1)	72%		5
Ruisenor Escudero et al. [71]	Afghanistan		2014	Cross-sectional			Community	548	40.3%		7
Nasir et al. [9]	Afghanistan		2010	Cross-sectional			Community	623	36%		7
Todd et al.[12]	Afghanistan		2011	Cross-sectional			Community	483	36.1%		7
Todd et al.[38]	Afghanistan		2007	Cross-sectional			Clinic	464	36.6%		8

**Table 5:** Key characteristics of included studies on co-infection of HIV and HCV

Author	Country	Year of Publication	Design	Setting	Sample Size	Co-infection of HIV and HCV	JBI Score
Alavi et al. [75]	Iran	2009	Cross-sectional	Hospital	142	8.5%	6
Davoodian et al. [23]	Iran	2009	Cross-sectional	Prison	249	14.5%	8
Hosseiniet al. [24]	Iran	2010	Cross-sectional	Detention center	417	24%	7
Javadi et al. [18]	Iran	2013	Cross-sectional	Drop in center	539	1.1%	9
Rahimi-Mofaghar et al. [16]	Iran	2010	Cross-sectional	Treatment center and Community	895	8.7%	8
Ramezani et al. [32]	Iran	2014	Cross-sectional	Clinic	100	15%	6
Sofian et al. [33]	Iran	2012	Cross-sectional	Detention center	153	5.2%	6
Zamani et al. [20]	Iran	2007	Cross-sectional	Drop in center and Community	202	9.4%	7
Alavi et al. [46]	Iran	2007	Cross-sectional	Hospital	154	50%	7
Honarvar et al. [74]	Iran	2013	Cross-sectional	Counseling center	233	6.4%	7
Achakzai et al. [37]	Pakistan	2007	Cross-sectional	Community	50	20%	3
Escudero et al. [71]	Afghanistan	2014	Cross-sectional	Community	548	6.8%	7
Nasir et al. [9]	Afghanistan	2011	Cross-sectional	Community	623	1.8%	7
Todd et al. [38]	Afghanistan	2007	Cross-sectional	Clinic	464	1.5%	8
Todd et al. yyy(12)	Afghanistan	2011	Cross-sectional	Community	483	1.7%	7

## Results

### Characteristics and quality of included studies

**Fig. 1:**
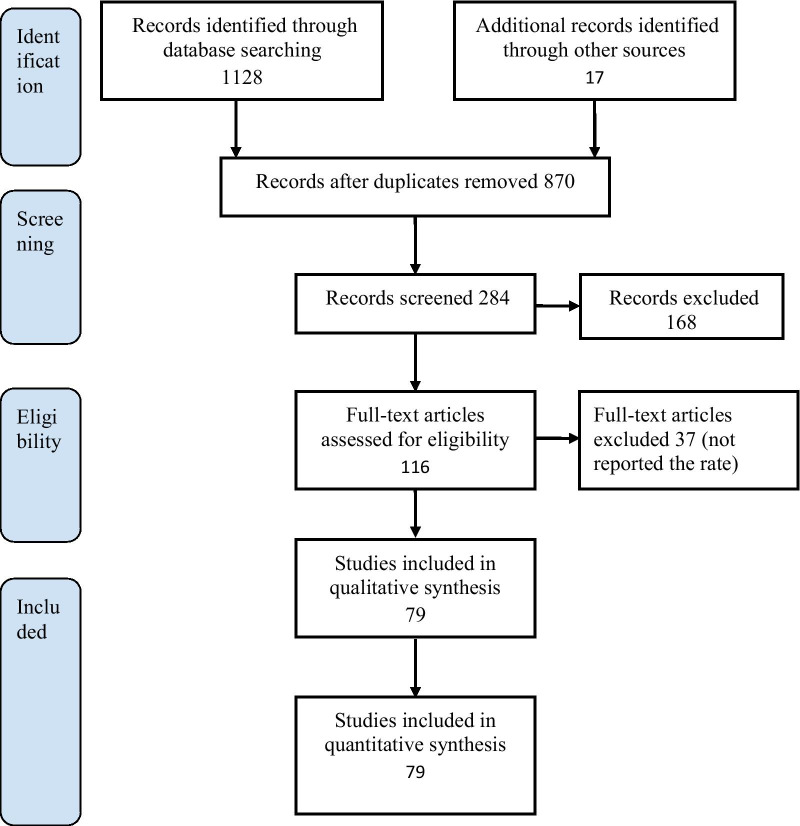
PRISMA flow diagram

In this review, 116 studies were screened, and 79 articles were included for data extraction (Fig. 1), 57 studies from Iran, 18 from Pakistan, and four from Afghanistan. The total number of participants in studies that assessed the prevalence of HIV among IDUs included 68,926 participants, while those from studies that assessed the prevalence of HCV were 23,016 participants. The study of Emmanuel (2013) contributed the highest number of participants (n = 46,351). Most studies identified were conducted in Iran. The majority of studies in Iran were carried out in non-community settings such as health centers and prisons. Those conducted in Pakistan and Afghanistan were carried out in community settings. All studies used a cross-sectional study design (Table 3 and 4). In Iran, the country-level analysis revealed that 17,261 IDUs were identified for HIV prevalence analysis while 17,894 participants were included in HCV analysis. In Pakistan, 49,547 IDUs were included in HIV studies, while 3004 participants were identified for the HCV prevalence analysis. In Afghanistan, a total of 2118 IDUs were included in both HIV and HCV prevalence analysis. Co-infection was reported in 15 articles with 5252 participants in three countries. Tables 3, 4, and 5 show the prevalence of HIV, HCV, and co-infection from studies identified in the three selected countries. The JBI assessment tool showed that 58 articles were with low risk of bias, 20 with moderate, and 1 with a high risk of bias (Table 3 and 4).

### Prevalence of HIV and HCV in Iran, Pakistan, and Afghanistan

Overall, HIV and HCV prevalence among IDUs in the three selected countries were 9.1% (95% CI 6.9–12.0%) and 48.3% (95% CI 43.9–52.7%), respectively. In country level analysis, Iran had the highest HIV prevalence among IDUs while Afghanistan had the lowest among three selected countries, 11.0% (95% CI 8.4–14.2%) and 3.1% (95% CI 1.5–6.3%), respectively. In Pakistan, the prevalence of HIV was 8.6% (95% 4.8–15.0%).

With respect to HCV prevalence, Pakistan had the highest prevalence while Afghanistan had the lowest, 54.4% (95% CI 33.5–73.9%) and 37.3% (95% CI 35.2–39.4%), respectively. HCV prevalence in Iran was 47.7% (95% CI 43.4–52.0%). Co-infection of HIV and HCV in three selected countries was 7.6% (95% CI 4.4–12.8%).

### Publication bias

There was no significant publication bias on HCV rate as shown by the result of the Egger test (*P* = 0.1), while there was publication bias on HIV rate (*P* = 0.001).

## Discussion

We present seminal evidence on the prevalence of HIV and HCV among IDUs in three selected countries in the EMRO-WHO region. These countries are in the neighborhood of each other. The pooled prevalence of HIV and HCV was assessed separately using country-level findings.

HCV prevalence rate was significantly high in the three selected countries; overall prevalence was 48.3% (95% CI 43.9–52.7%). We report an overall average HIV prevalence of 9.1% (95% CI 6.9–12.0%). We acknowledge the fact that most studies have been done mainly in Iran. Indeed, a review of HIV among IDUs in the Middle East and North Africa also indicated that only Iran had a substantial number of studies on this subject [14]. Nonetheless, similar studies have reported a higher prevalence of HIV in Iran 15% [5–25], Pakistan 10.8% (9.6–12.1), and Afghanistan 3.4% (1.7–5.1) [15]. These estimates are similar to the findings of our review.

Another systematic review that sought to estimate the global prevalence of HCV infection among IDUs revealed that HCV prevalence among IDUs in Pakistan ranged from 78 to 93%, while that in Iran ranged from 54.9 to 80.1% [11]. This is inconsistent with our finding primarily due to differences regarding sources of information that were used. This could be attributed to the time difference and grey literature considered in the former review. Those studies were done a decade ago, and also substantial numbers of grey literature and experts' views were included in the analysis. Noteworthy, in our study, we used only peer-reviewed articles; however, the former reviews considered various sources, including peer-reviewed papers, reports from relevant organizations, books and booklets, slides, press articles, and personal communiqués. These provide a broad scope of findings; however, they are liable to systematic bias associated with such designs.

The large range estimates reflect uncertainty about estimates derived from these studies. Secondly, the time difference between these studies may affect the results.

The variability among countries in terms of the number of studies that reported prevalence of HIV and HCV among IDUs might be due to varied research capacity across the three selected countries and varying investment in research capacity building. This issue may be a basis to initiate collaborations aimed at capacity building. Moreover, it could serve as a turning point and priority for tackling various public health challenges.

Regarding the geographical scope of the studies, nearly all of the included articles in our review were from the largest cities of these three selected countries. This is in one way reflective of the availability of resources in these cities and, on the other hand, imbalanced research attention to countrysides. The Iceberg term can be used for this situation due to those geographic areas that are deprived of resources. In order to have a clear picture of the situation, we need studies with larger sample sizes. These should also include different geographical areas.

The available evidence has emphasized that HIV and HCV infections represented a major adverse health consequence among IDUs. This causes a considerable health burden in this region. Our review is the first research of its kind that illustrates HIV and HCV rates among IDUs in the context of three neighboring countries that are highly implicated in drug smuggling and production globally. Comprehensive public health interventions are required to address this problem nationally and internationally.

However, the limitation of the study should be considered in the time of using the findings, in which grey literature was not included in the study. Due to the low capacity of research, especially in Afghanistan, this could influence the result and hide the actual rate of HIV and HCV among IDUs.

## Conclusions

Our review revealed that the prevalence of HIV and HCV is significantly high among IDUs in Iran, Afghanistan, and Pakistan. Injecting drug users are at high risk of HIV and HCV transmission and can spread infections to the community due to unhealthy behavior, including syringe change and unsafe sex. Therefore, interventions are required at different levels of prevention for this high-risk group.

## Data Availability

Not applicable for this study.

## Abbreviations

HIV: Human immunodeficiency virus
HCV: Hepatitis C Virus
IDUs: Injecting drug users
WHO-EMRO: World Health Organization-East Mediterranean Regional Office
